# The awareness and practice of testicular self-examination among male undergraduates in Nigeria: a descriptive cross-sectional study

**DOI:** 10.1186/s12909-022-03562-w

**Published:** 2022-06-25

**Authors:** Bukola Mary Ibitoye, Eniola Khadijah Suleiman, Ama Gyamfua Ampofo

**Affiliations:** 1 Department of Nursing Science, College of Health Sciences, University of Ilorin, Ilorin, Kwara State Nigeria; 2 Health Behaviour Research Collaborative, Priority Research Centre for Health Behaviour, School of Medicine and Public Health, University of Newcastle, Callaghan, Australia

**Keywords:** Testicular self-examination, Undergraduates, Testicular cancer, Healthcare education, Nigeria

## Abstract

**Background:**

Testicular cancer is a relatively rare form of cancer but curable. In Nigeria, late presentation hinders treatment due to limited resources for diagnosis and treatment. Testicular self-examination enables men to identify the presence of lumps and any abnormality in their testes. This can facilitate early detection and presentation at hospitals. The purpose of this study was to examine the awareness and practice of testicular self-examination by students at a College of Health Sciences in a Nigerian university.

**Methods:**

A cross-sectional study was conducted. The target population were second-sixth year students in the College of Health Sciences. The respondents were conveniently selected to complete a 38-item, self-administered questionnaire. The paper-based questionnaire was distributed to 280 respondents in classrooms and dormitories. Descriptive statistics (such as percentages and frequencies) were used to summarize the frequency of categorical data.

**Results:**

Of the 277 respondents, only 53.4% (*n* = 148) have heard about testicular self-examination. The mean age was 20.6 (± 4.51) years. Out of the 148 respondents, only 11.6% practiced it regularly. For majority of the respondents, the barriers mitigating the practice of testicular self-examination are the fear of discovering a lump and lack of knowledge.

**Conclusion:**

It is necessary for the importance of testicular self-examination to be emphasized in the training of nurses, medical doctors, and other healthcare professionals and its practice should be encouraged among health science students. This will equip these students with the knowledge and skills for their health and to educate their patients and the society on the relevance of testicular self-examination.

**Supplementary Information:**

The online version contains supplementary material available at 10.1186/s12909-022-03562-w.

## Introduction

Testicular cancer (TC) is a rare form of cancer [1]. It is estimated that about 8,000–10,000 new cases are diagnosed each year globally. TC has a very high cure rate of about 95%; it is estimated that only 400 deaths will be recorded from TC yearly in the USA [2, 3]. Despite its rarity, it is the most common cancer affecting men between the ages of 15 – 49 years; and it is the second most common cancer affecting men aged 15–19 years globally [4]. The mean age for a diagnosis of TC is 33 years [5]. However, TC can occur at any age. Over many decades, the incidence rate of testicular cancer in several countries has been increasing [3]. The highest incidence rates were recorded in Western and Northern European countries and the lowest is recorded in Africa [6]. Retrospective studies have shown that the prevalence rate of TC is low in Nigeria [7, 8]. A prospective study of 24 patients showed that five died, five survived beyond five years post-treatment, and the rest were lost to follow up and did not complete treatment [9]. Of these patients, 62.9% reported when the disease was at Stage 3 or 4. This study exemplifies the challenges associated with TC management in Nigeria [9]. Despite the low prevalence rate, TC management is still hindered by late presentations and limited resources for cancer prevention, diagnosis, and treatment [9].

Although TC is a highly curable disease, successful treatment is dependent on early diagnosis of the condition [2]. If treatment is not commenced immediately, it spreads rapidly to other body parts. Hence, it is imperative that the disease is diagnosed promptly. Some types of TC are asymptomatic until they reach an advanced stage; however, most TC manifest with the presence of a small lump which can be easily detected through testicular self-examination (TSE) before the disease spreads [10]. TSE is the inspection of the appearance and texture of testicles to observe their condition and help detect when abnormal changes occur [11]. While some experts dispute the relevance of TSE, others believe it can be helpful in the early detection of TC [10]. It is often recommended that men at risk of TC perform TSE monthly, but some doctors recommend that TSE be performed monthly after puberty by all men [10].

The TSE should be performed after a hot bath/shower because the testes are more relaxed; hence, the testicles can be easily palpated. During TSE, the man should look out for the following: the presence of smooth or rounded lumps and changes in size, shape or consistency of the testes [10]. TSE can also help in the early detection of testicular torsion and varicocele [12]. Hence, it is imperative that men perform TSE regularly to aid early detection of TC and other abnormalities.

Some studies have explored the awareness of TC and practice of TSE among various groups of teenage and adult men. Some studies in Nigeria found that most had good knowledge about TSE but never practised it [13, 14]. Other studies reported a poor awareness about TC and TSE and low practice of TSE [15–17]. Another study reported a deficit in men's awareness towards TSE, and they reported that less than one in five men regularly examine their testicles [18]. Respondents' reasons for not performing TSE include feeling embarrassed by TSE, lacking the knowledge and feeling incompetent, and not caring about TSE [13, 15, 16]. Studies focussing on medical and nursing students have shown a high level of awareness, low knowledge of both TC and TSE, and poor practice of TSE [16]. It appears that many men do not understand the risks of TC and the importance of TSE [19]. This shows that possessing knowledge does not always translate to a regular practice of TSE. These studies have recommended the use of various educational strategies to increase awareness about TC and the importance of TSE. Similar strategies have successfully improved the awareness of breast and cervical cancer, the practice of breast self-examination and the uptake of cervical cancer screening strategies [20–22].

This study examined the awareness and practice of TSE by male undergraduate students at a College of Health Sciences. This is important in health promotion and disease prevention particularly for their prospective patients when they commence their practice as health practitioners. As future healthcare professionals, they play an essential role in educating patients and the society on relevant preventive strategies against diseases such as TC. However, if they are not knowledgeable about TSE then they will be ill-equipped to provide this education. This study will provide lecturers and heads of nursing, medical and allied health schools the opportunity to identify the deficiencies in the educational system regarding TSE to establish/update policies and courses to ensure TSE is included in the training of these future healthcare professionals. Though this study was conducted in Nigeria, its findings can influence medical and nursing education globally by drawing the attention of lecturers and faculty members to the importance of including TSE in the training of healthcare professionals. Also, this study will provide baseline information that will guide the development of interventions and serve as reference material for men, teachers, and researchers. The objectives of this study were to determine the awareness and practice of TSE among male undergraduate students in a Nigerian university, and to explore any association between awareness and practice of TSE.

## Methods

### Study design, setting and sampling

This study conducted a descriptive cross-sectional study survey to achieve the research aim. The study was conducted at the College of Health Sciences, University of Ilorin, Nigeria. At the time, this research was conducted the college ran five undergraduate programs with different durations, they are: Bachelor of Medicine and Bachelor of Surgery (MBBS) (6 years), Bachelor of Nursing Science (5 years), BSc. Anatomy (4 years), BSc. Physiology (4 years) and BSc. Physiotherapy (5 years). The students from BSc. Physiotherapy was excluded because their first batch of students were in their first year. First year students at the university do not take programme-specific healthcare courses hence they were excluded from the study. The respondents were male undergraduates at the College. This study was conducted between August and November 2019.

The respondents were conveniently selected. The inclusion criterion was any male undergraduate in 2^nd^—6^th^ year of study. The exclusion criterion was undergraduates who were not present when data collection were ongoing.

### Data collection procedure

A self-administered questionnaire, developed by the researchers, was used as the data collection instrument. The questionnaire was developed by reviewing previous studies on this topic [13, 23]. The paper-based questionnaire contains 38 questions which was divided into four sections. Section A was designed to reveal the socio-demographic data of respondents; Section B, C, D and E contained questions on awareness of TSE, the practice of TSE, knowledge of the steps of TSE, and factors influencing the practice of TSE.

The questionnaire was pre-tested among 20 respondents at a different college. This was done to assess the reliability of the questionnaire; after analyzing the results, the necessary corrections were made. Data collection was done in October 2019. See the questionnaire in supplementary material 1. The data was analyzed with IBM® Statistical Package for Social Sciences (SPSS) (version 20.0). Descriptive statistics was used to describe the categorical data. Chi-square was calculated to determine the association between awareness and practice of TSE and p-value less than 0.5 were considered significant.

The questionnaires were distributed and collected by one of the authors from 1st to 14th October 2019. The researcher approached eligible students in dormitories and classrooms. She explained the study aims to eligible students and obtained informed consent from students who were willing to participate. Ethical permission was obtained from the Department of Nursing Science. To ensure confidentiality, no identifying information was requested from the respondents, and they were informed to avoid writing any form of identification on the questionnaires. All the completed questionnaires were put in an envelope, sealed, and placed in a secured compartment.

## Results

A total of 280 questionnaires were distributed, 277 were retrieved. The mean age was 20.6 ± 4.51 years and variance of 20.37. The highest proportion of the respondents were medical students (59.2%), Christians (58.5%) and in their 4^th^ year in the university (45.8%). The characteristics of the respondents are shown in Table 1.

**Table 1:** Socio-demographic data of respondents

Variable	Category	Frequency (%) (*n* = 277)
**Age (years)**	16–20	185 (67)
	21–25	60 (22)
	26–30	9 (3)
	31–35	23 (8)
**Religion**	Christianity	162 (59)
	Islam	115 (42)
**Level**	200	17 (6)
	300	92 (33)
	400	127 (46)
	500	41 (15)
**Relationship status**	Single	182 (66)
	Married	32 (12)
	Dating	63 (23)
**Course of study**	Medicine	164 (59)
	Anatomy	57 (21)
	Nursing	40 (14)
	Physiology	16 (6)

### Awareness of TSE

Majority of the respondents (*n* = 148, 53%) have heard about TSE; 47% (*n* = 129) had never heard of TSE. Among those who had heard about TSE, 89% (*n* = 132) believed it could help in early diagnosis of testicular cancer; the majority (67%, *n* = 98) believed a man should start TSE between 16–20 years of age, 22% indicated 21–25 years, 3% indicated 26–30 years, and 8% indicated 31 years and above.

### Practice of TSE

The majority, 72% (200) of the respondents, have never performed TSE before. Only 28% (*n* = 77) practiced TSE, but only 12% (n = 32) practiced it regularly. Less than one-third (24.9%, *n* = 69) knew the steps involved in performing TSE. Only 16% (*n* = 44) perform TSE monthly; 10% performed weekly; 2% (*n* = 5) did it once a year. A low percentage (16%, *n* = 43) admitted that TSE was time-consuming. An even lesser percentage (*n* = 36, 13%) indicated that it was difficult to perform. The 148 respondents (53%) who were aware of TSE were asked to complete questions assessing their knowledge on the steps involved in performing TSE. Table 2 shows the respondents that accurately answered the questions. Table 3 shows the factors that influence the respondents' practice of TSE. For the section, respondents were encouraged to select multiple responses. The fear of discovering a lump influences the practice of TSE in 69% (*n* = 190) of the respondents. A large majority (*n* = 210, 76%) admitted that lack of knowledge influences their practice. There was a significant association between awareness of TSE and its practice (X^2^ = 8.524, *p* = 0.004).

**Table 2:** Steps in performing testicular self-examination

Questions	Correct Response	Frequency (%) (*n* = 148)
*Correct statements*		
Stand in front of the mirror and look for swelling on the scrotum	Yes	118 (80)
Using both hands the scrotum should be gently lifted so that the area underneath can be checked	Yes	54 (37)
The index and the middle finger should be placed under each testicle with the thumb on top	Yes	117 (79)
The testes should be examined one at a time	Yes	123 (83)
Roll each testicle between fingers and thumb	Yes	108 (73)
Feel for lumps of any size (even as small as a pea) in the testicle	Yes	124 (84)
*Incorrect statements*		
Lie on the bed and look for swelling on the scrotum	No	63 (43)
Use both hands to examine both testes together as one	No	75 (51)
Roll each testis with thumb alone	No	64 (43)

**Table 3:** Barriers and facilitators influencing the practice of TSE

S/N	Questions	Frequency (%)
1.	TSE plays a major role in the early detection of testicular cancer	120 (81)
2.	Lack of knowledge on how to do TSE	112 (76)
3.	I am afraid of discovering a lump	102 (69)
4.	Touching my testes is embarrassing	55 (37)
5.	Doing TSE is time-consuming	45 (30)
6.	Touching my testes is a sin	22 (15)

Respondents were allowed to choose more than one response

## Discussion

With the rising incidence of TC, it is crucial that men regularly practice TSE to aid early detection and cure of TC. Our study highlights the awareness and practice of TSE and the barriers and facilitators affecting its practice among undergraduates of a College of Health Sciences. Our study shows that most of the students had heard of TSE; however, a significant minority (46%) had not heard about TSE. For health science students, this is not an impressive percentage. However, this figure is higher than the 32% of 225 students reported in a study in Ethiopia among health science students [19]. A study in Uganda among secondary school students reported that 58% of 165 students had never heard about TSE [24]. A similar study in a Nigerian secondary school reported that 98.7% of 540 students had never heard about TSE [25]. Undoubtedly, it is expected that health science students would have heard about TSE, compared to secondary school students. A conclusion on the comparison between the two groups is beyond the scope of this study. However, it is evident that there is inadequate sensitization about TSE among young males in our study area. This may be related to the fact that education on TSE and TC is inadequate during medical and nursing education. The inadequacy may be due to the rarity of TC. Consequentially, these students will not be able to educate their patients and peers about the importance of TSE, and this will further worsen the level of awareness of TSE in the general public. Hence, there is an urgent need for health science students to be educated on TSE as part of their curriculum if not present.

The majority of the respondents indicated that young men should start performing TSE between the ages of 16–20 years. This is a positive finding. With the average age at the time of diagnosis of testicular cancer being 33 years, TC is primarily a disease of young and middle-aged men. Though about 6% of cases occur in children and teens, and about 8% occur in men over the age of 55 [5]. Therefore, it is essential that adolescents and young men are encouraged to practice TSE and educate their peers. Also, young men ought to be encouraged to determine their level of risk of developing TC. This will aid early detection and increase their chances of surviving TC.

Less than one-third of the respondents practice TSE and a smaller fraction (11.6%) practice it regularly. This result is similar to the findings of other studies [13, 14, 16, 17, 19]. In this current study, one in ten men practice TSE regularly. This is lower than the finding reported in a study, where less than one in five men regularly performed TSE [18]. Majority of the respondents indicated that lack of knowledge of the process of performing TSE is the reason for the low practice. This low practice implies that TC may not be discovered early hence increasing the mortality rates of the disease. Three major factors were identified as barriers to the practice of TSE among the respondents. They are fear of discovering a lump, lack of knowledge on how to perform the TSE and belief that older men should perform it. A systematic review involving 25 studies reported similar findings where the fear of detecting a lump played a significant role in the refusal to perform TSE [26]. It is important to emphasize to young men that TC is a highly curable disease hence detecting it early greatly improves their chances of survival.

Another relevant finding in this study is that the lack of knowledge on TSE is a major factor mitigating the practice of TSE. Similar findings were reported in Turkey and Nigeria among high school and tertiary students who had inadequate education of TSE [25, 27]. A possible explanation for these findings could largely be attributed to the lack of practical understanding of TSE in Nigeria. The significant number of health students who have never heard of TSE is a great worry and should be of great concern since their knowledge about TSE cannot be translated into practice. Students in health sciences are placed in hospitals for internships or placement and thus form part of the health team, and during these times, they are involved in patient care, education, and a point of contact for information for most patients. Interestingly, most of these students are in their final year and should have covered TSE in school. Hence, the need to revisit the curriculum and incorporate practical approaches to delivering courses studied by this group of students. It is also possible that TSE may have been taught as a part of a course; however, students could not understand it, or they opted to subscribe to the 'learn, pass and forget' cliché, which may have contributed to this finding. Thus, it is necessary to adopt new approaches to teaching and learning to enhance understanding.

The current study provided insights into the practice of TSE and factors influencing its practice. However, the study took place in a single institution with a sample size which were conveniently sampled, hence may not generalized to the whole Nigerian health science students' population. Hence, the results should be interpreted with caution. This study did not identify the sources of information regarding TSE, future studies should investigate this because it can guide the development of awareness campaigns. Future studies should look at multiple sites and a larger sample size representative of the population in Nigeria. We believe that our findings can form a basis for multicenter studies.

## Conclusion

The knowledge and practice of TSE are very low, which could be attributed to lack of education, fear of detecting a lump and the belief that the older men should perform TSE, among health science students in the University of Ilorin, Nigeria. We recommend that educational interventions on TSE should be developed to address these misconceptions. Nurses, doctors, and other health educators in Nigeria and other countries can utilize learning materials such as leaflets and mobile applications to enhance understanding of TSE. For instance, the process of TSE and what happens during a doctor’s appointment after a lump detection can be taught through a cartoon video or a poster. This will encourage men to perform regular TSE. Also, the curriculum for nursing and medical students in the universities pertaining to TSE should be introduced early using appropriate teaching methods to enhance understanding. 

## Supplementary Information

**Additional file 1. **Questionnaire.

## Data Availability

The datasets analysed during the current study are available from the corresponding author on reasonable request.

## Abbreviations

TC: Testicular cancer
TSE: Testicular self-examination
