# Supplementary material for: The awareness and practice of testicular self-examination among male undergraduates in Nigeria: a descriptive cross-sectional study
Source: BMC Med Educ. 2022 Jun 25;22:495. doi: 10.1186/s12909-022-03562-w (PMC9233382; doi:10.1186/s12909-022-03562-w)
Supplement: Supplementary file 1 — Additional file 1. Questionnaire. [file 12909_2022_3562_MOESM1_ESM.docx]

**Questionnaire**

**Title: The awareness and practice of testicular self-examination among male undergraduates in Nigeria:**  a descriptive cross-sectional study

Ibitoye, Bukola Mary^1^, Suleiman, Eniola Khadijah^1^, Ampofo, Ama Gyamfua^2^

1. Department of Nursing Science, College of Health Sciences, University of Ilorin, Ilorin, Kwara State, Nigeria
2. Health Behaviour Research Collaborative, Priority Research Centre for Health Behaviour, School of Medicine and Public Health, University of Newcastle, Australia

**Section A. Socio-demographic information**

*Please write or tick (√) as appropriate*

1. Age: ……….
2. Religion: Christianity [ ] Islam [ ] Traditional [ ] Other [ ],

Please specify…………..

1. Course of study: Anatomy [ ] Medicine [ ] Nursing [ ] Physiology [ ]
2. Level of study: 200 [ ] 300 [ ] 400 [ ] 500 [ ] 600 [ ]
3. Relationship status: Single [ ] Married [ ] Dating [ ] Engaged [ ]

**Section B. Awareness of Testicular Self-Examination**

*Select the most appropriate answer for the following questions on your awareness of Testicular Self-Examination*

1. Have you ever heard of testicular self-examination? Yes[ ] No [ ]

**Proceed to Question 2 if your answer is Yes**

1. Who should know about testicular self-examination? All men [ ] Male students[ ] Health workers[ ] Other [ ], Please specify………………..
2. Does testicular-self-examination help in the early diagnosis of testicular cancer? Yes[ ] No[ ]
3. Is awareness of testicular self-examination important to men? Yes[ ] No [ ]
4. At what age should one start testicular self-examination? 16-20[ ] 21-25[ ] 26-30[ ] 31and above[ ]
5. When is the best time to perform testicular self-examination? Every week [ ] every month[ ] once in a year[ ] I don’t know[ ]

**Section C. Practice of testicular Self- Examination**

*Select the most appropriate answer for the following questions on your practice of Testicular Self-Examination*

1. Do you practice Testicular Self-Examination? Yes [ ] No[ ]
2. Do you know the process and steps involved in Testicular Self-Examination?

Yes[ ] No[ ]

1. When do you practice Testicular Self-Examination? Every week [ ] Every month[ ] Once in a year[ ] I don’t know[ ]
2. Is Testicular Self–Examination time-consuming? Yes[ ] No[ ]
3. Do you practice Testicular Self –Examination regularly? Yes[ ] No[ ]
4. Do you feel satisfied with the way you perform Testicular Self –Examination? Yes[ ] No[ ]
5. How would you rate your performance? Excellent [ ] Very good[ ] Good[ ] Fair [ ] Poor[ ]
6. Should Testicular Self–Examination be practiced by only people at risk of testicular cancer? Yes[ ] No[ ]
7. Is Testicular Self–Examination difficult to perform? Yes[ ] No[ ]
8. How often do you think Testicular Self–Examination should be performed?

Daily[ ] Weekly[ ] monthly[ ] annually[ ] when a lump is suspected[ ] I don’t know[ ]

1. When last did you perform Testicular Self–Examination? Weeks ago [ ]Months ago[ ] One month[ ] A year ago[ ] Never[ ]

**Section D: Steps for performing** **Testicular Self –Examination**

*Answer the following questions regarding the steps involved in performing testicular self-examination.*

*If the statement is correct, select Yes; if it is incorrect, select No*

1. Stand in front of the mirror and look for swelling on the scrotum Yes[ ] No[ ]
2. Lie on the bed and look for swelling on the scrotum Yes[ ] no[ ]
3. Using both hands, the scrotum should be gently lifted so that the area underneath can be checked Yes[ ] No[ ]
4. The index and the middle finger should be placed under each testicle with the thumb on top. Yes[ ] No[ ]
5. The testes should be examined one at a time Yes[ ] No[ ]
6. Use both hands to examine both testes together as one Yes[ ] No[ ]
7. Roll each testis with thumb alone Yes[ ] No[ ]
8. Roll each testicle between fingers and thumb Yes[ ] No[ ]
9. Feel for lumps of any size Yes[ ] No[ ]

**Section D. Factors influencing the practice of testicular Self –Examination**

*Select the factors influencing your practice of Testicular Self –Examination.*

*Select all that apply*

1. Touching one’s testes is embarrassing? Yes [ ] No[ ]
2. Fear of discovering a lump? Yes[ ] No[ ]
3. Lack of knowledge on how to do Testicular Self–Examination? Yes[ ] No[ ]
4. Touching one’s testes is a sin? Yes[ ] No[ ]
5. Testicular Self–Examination plays a major role in early detection of testicular cancer? Yes[ ] No[ ]
6. Doing Testicular Self–Examination is time-consuming? Yes[ ] No[ ]
7. Testicular Self–Examination should be done by older men? Yes[ ] No[ ]
